# Data Management and Data Quality in PERCH, a Large International Case-Control Study of Severe Childhood Pneumonia

**DOI:** 10.1093/cid/cix080

**Published:** 2017-05-27

**Authors:** Nora L. Watson, Christine Prosperi, Amanda J. Driscoll, Melissa M. Higdon, Daniel E. Park, Megan Sanza, Andrea N. DeLuca, Juliet O. Awori, Doli Goswami, Emily Hammond, Lokman Hossain, Catherine Johnson, Alice Kamau, Locadiah Kuwanda, David P. Moore, Omid Neyzari, Uma Onwuchekwa, David Parker, Patranuch Sapchookul, Phil Seidenberg, Arifin Shamsul, Kazungu Siazeele, Prasong Srisaengchai, Mamadou Sylla, Orin S. Levine, David R. Murdoch, Katherine L. O’Brien, Mark Wolff, Maria Deloria Knoll

**Affiliations:** 1 Emmes Corporation, Rockville, and; 2 Department of International Health, International Vaccine Access Center, Johns Hopkins Bloomberg School of Public Health, Baltimore, Maryland;; 3 Milken Institute School of Public Health, Department of Epidemiology and Biostatistics, George Washington University, Washington, District of Columbia;; 4 Department of Epidemiology, Johns Hopkins Bloomberg School of Public Health, Baltimore, Maryland;; 5 Kenya Medical Research Institute–Wellcome Trust Research Programme, Kilifi;; 6 International Centre for Diarrhoeal Disease Research, Bangladesh (icddr,b), Dhaka and Matlab;; 7 University Teaching Hospital, Lusaka, Zambia;; 8 Medical Research Council, Respiratory and Meningeal Pathogens Research Unit,; 9 Department of Science and Technology/National Research Foundation, Vaccine Preventable Diseases Unit, and; 10 Department of Paediatrics and Child Health, Chris Hani Baragwanath Academic Hospital and University of the Witwatersrand, Johannesberg, South Africa;; 11 Centre pour le Développement des Vaccins (CVD-Mali), Bamako;; 12 Medical Research Council Unit, Basse, The Gambia;; 13 Global Disease Detection Center, Thailand Ministry of Public Health–US Centers for Disease Control and Prevention Collaboration, Nonthaburi;; 14 Center for Global Health and Development, Boston University School of Public Health,Massachusetts;; 15 Department of Emergency Medicine, University of New Mexico, Albuquerque;; 16 Bill & Melinda Gates Foundation, Seattle, Washington;; 17 Department of Pathology, University of Otago, and; 18 Microbiology Unit, Canterbury Health Laboratories, Christchurch, New Zealand

**Keywords:** data management, electronic data capture, data quality; PERCH.

## Abstract

The Pneumonia Etiology Research for Child Health (PERCH) study is the largest multicountry etiology study of pediatric pneumonia undertaken in the past 3 decades. The study enrolled 4232 hospitalized cases and 5325 controls over 2 years across 9 research sites in 7 countries in Africa and Asia. The volume and complexity of data collection in PERCH presented considerable logistical and technical challenges. The project chose an internet-based data entry system to allow real-time access to the data, enabling the project to monitor and clean incoming data and perform preliminary analyses throughout the study. To ensure high-quality data, the project developed comprehensive quality indicator, data query, and monitoring reports. Among the approximately 9000 cases and controls, analyzable laboratory results were available for ≥96% of core specimens collected. Selected approaches to data management in PERCH may be extended to the planning and organization of international studies of similar scope and complexity.

The Pneumonia Etiology Research for Child Health (PERCH) is a multisite study to estimate the etiology of childhood pneumonia in low- and middle-income countries [1, 2]. To meet study objectives, PERCH required up to 12 different clinical forms and up to 17 different laboratory forms (average 16 clinical and laboratory forms submitted per case or control). For a given case, the total number of potential data fields exceeded 3000. In anticipation of the volume and complexity of data to be collected, the PERCH Core team located at the Johns Hopkins Bloomberg School of Public Health (JHSPH) partnered with the Emmes Corporation (Rockville, Maryland) to serve as the data coordinating center (DCC) and to provide supplemental statistical support. Data management strategies aimed to meet technical and operational requirements of the standardized study protocol implemented across diverse research sites. The DCC, PERCH Core team, and field sites collaborated to design a system in which accumulating data could be monitored and analyzed in near real-time. In this way, deviations from standard study procedures could be resolved rapidly and analyses could begin while the study was under way.

In this article we describe our data management strategies and evaluate their value and challenges.

## DATA MANAGEMENT STRUCTURE AND IMPLEMENTATION

### Core Team, Field Site, and DCC Structure

The JHSPH PERCH Core team led the overall management of study operations and communications among field site principal investigators (PIs), field site staff, DCC, and sponsor. The Core team consisted of the overall study PI, lead investigators in 4 content areas (data management/analysis, clinical/epidemiology, clinical metrics standardization, and laboratory), a senior and junior statistician, and 2–4 full-time study coordinators/analysts, depending on the stage of the study. The Core team engaged with sites and external experts to develop the study protocol, case report forms (CRFs), standardization materials, and standard operating procedures (SOPs) (available for download at: http://www.jhsph.edu/research/centers-and-institutes/ivac/projects/perch/). Materials were piloted at each site and as part of a dedicated pilot study at the Kilifi site [3]. Sites did not start formal data collection until they had successfully completed a pilot phase that lasted approximately 3 months and included a review of the CRFs, electronic data capture (EDC) data entry practice, and/or enrollment of pilot cases or controls (duration and activities for pilot phase varied by site). The Core team visited sites during the planning/preparation phase, at study initiation, and biannually or annually to monitor protocol adherence. The visiting team included members with expertise in laboratory, clinical, and epidemiological methods as well as study coordinators and data managers. Regular multisite teleconferences reinforced training in standard methods, identified challenges as they arose, and recommended strategies for efficient workflow.

Each field site had dedicated data management staff, a study coordinator, and clinical and laboratory leads. Between 13 and 37 clinical staff per site were trained in case and control evaluation [4]. Over the course of the study, an average of 30 staff members per site were trained to enter or update data in the data system; at any one time approximately 2–10 staff per site were performing regular data entry.

The DCC was comprised of a PI (statistician or epidemiologist), several data managers, a SAS programmer, and a database developer with information technology (IT) support. The DCC PI was responsible for the overall management of DCC activities, quality assurance of the PERCH data system, and operational and analysis reports prepared by the DCC. In collaboration with the JHSPH Core Team, the DCC designed electronic versions of the CRFs (eCRFs), key data checks, and data quality reports, and maintained a central study website for training and communications.

### Electronic Data Capture System Development

A centralized EDC system was implemented to ensure standardization of data. This electronic system minimized the logistical challenges of transporting, managing and storing large volumes of paper CRFs. The DCC, with an established EDC system and large data management and analytic capacity, was chosen to accommodate both aggressive study timelines and rigorous quality requirements. The DCC tailored their EDC system to guide data collection according to the PERCH protocol and schedule of procedures. The system applied detailed value and range checks within and across forms. Entered data triggered system logic that enabled or prevented access to certain fields or forms, directing data entry staff to applicable fields. Built-in, real-time missing form and missing field reports summarized overdue and incomplete data. The EDC system was adapted to site-specific conditions, and certain response values, fields, or forms were available only at sites where that information was applicable (eg, malaria and human immunodeficiency virus [HIV] testing only at select sites).

Minor modifications to the CRFs were required during the study. The internet-based system facilitated rapid implementation across sites and prevented old versions from mistakenly being used. Each version of the EDC system was tested before releasing to the live, study data system. A cumulative summary of system changes was maintained on the study website.

One site (Kilifi, Kenya) did not use Emmes’ EDC system to collect PERCH data, to prevent disruption of an existing system used to collect data across multiple interlinked studies. To standardize the Kenya dataset for PERCH, the Kenya data manager mapped data structures to the PERCH EDC format, deriving new or recoded fields to match characteristics of PERCH SAS tables. This complex programming avoided redundancy and the substantial effort needed to adopt an alternative PERCH EDC at the Kenya site. At intervals throughout the study, the DCC verified format and appended the standardized Kenya datasets to PERCH-wide datasets where data cleaning, monitoring, and analyses were performed as for the other sites.

Two sites (Mali and Thailand) required the EDC system to display both English and local languages. CRFs were translated from English to French or Thai by site staff. Translations were evaluated by lead investigators at each site to confirm replication of the English interpretation. Final translations were programmed by the DCC to appear in eCRFs.

### Internet-Based Data Entry System

Another key decision for the PERCH study was to use an internet-based EDC system. This decision was made to allow (1) monitoring of data entry and quality in near real-time so that patterns of missing data and data errors could be swiftly identified and resolved; (2) monitoring of study operations in real time (eg specimen volume and time from specimen collection to receipt in laboratory) so solutions could be quickly implemented when needed; (3) entry of data at multiple locations of collection at each site (eg, laboratory, clinic, emergency room); and (4) quick implementation of any required CRF changes.

Site capacity to implement an internet-based EDC system was evaluated by internet speed tests conducted by the DCC and through a questionnaire administered to the sites on power supply and internet reliability, available hardware, and language requirements. Results indicated potential to adopt the EDC system at main site facilities where internet service was reliable or had infrequent interruptions.

### Data Capture

#### Clinical Data

Entry of clinical data occurred at the location where cases were enrolled. Several sites (The Gambia, Mali, and Zambia) preferred direct data entry into the EDC system because of the built-in skip logic, prompts, and immediacy of identifying errors or missing data that could be addressed by staff in real time. However, this approach relied on a consistent internet connection and clinic staff who were comfortable with EDC. During periods of poor internet connection, these sites used paper CRFs until the data could be entered in the system. Where direct data capture was deemed less efficient or impractical due to slow or unreliable local internet, initial paper-based data collection was preferred. At these sites, data recorded on paper forms were entered into the EDC system by dedicated clinical, laboratory, and/or data entry staff. For some sites, collecting data on paper CRFs allowed staff to more easily verify the study data against surveillance data or hospital records.

To enroll controls from the community or at HIV clinics where there was often little or no internet access, most sites used paper CRFs that were returned to the main site for entry the same day. In Mali and Kenya, field workers enrolling controls in the home entered data directly on laptops that were either equipped with wireless cards or synced when they returned to the study facility.

#### Identification Numbers

All paper forms, log books, and specimen collection containers were labeled with barcodes that denoted the child/specimen study identification (ID) number; whenever possible, these were scanned into the EDC system to prevent transcription errors. Barcodes for stored specimens and isolates were printed on polyester cryolabels (Partnered Print Solutions) with adhesive designed to withstand freezing at –196°C.

#### Specimen Tracking

At 5 of the 7 sites, specimen inventories were managed using FreezerPro (Ruro, Frederick, Maryland), an internet-based system that allowed site laboratories to efficiently track storage and shipments to the central laboratory. Two other sites (Kenya and The Gambia) used existing local specimen databases. Maintaining electronic specimen inventories allowed the site laboratory staff to more easily manage a complex testing algorithm including batch polymerase chain reaction (PCR) testing, track the movement of samples between local laboratories, and prepare shipments to the central study laboratory.

#### PCR Results

PCR results in the form of electronic data sheet (.eds) output files were generated automatically by the ABI 7500 thermocycler (Thermo Fisher Scientific), for each plate run. These results were exported to a standardized comma separated values (.csv) template where they provided sample ID, cycle threshold value, and quantification data for each PCR target. The .eds and.csv PCR results files were transferred to the Core team approximately monthly by site laboratory staff via secure file transfer, preventing risk of transcription error. Following a technical review by the Core team, the files were transferred to the DCC, who used SAS programs to identify discrepant and out of range results, and convert final, validated results files to analyzable SAS datasets. This approach required review of the format for each results file for consistency to ensure compatibility with the standard importing format.

#### Transfer and Evaluation of Chest Radiographic Images and Digital Chest Auscultation Sound Files

Chest radiographs (CXRs) were collected as digital images at 5 sites and, at 2 sites (Zambia and Matlab), as analog images that were scanned into digital images [5]. CXR images were transferred monthly to the DCC in an automated process that embedded each image within an eCRF used to capture the interpretation of that image. The DCC designed the EDC system to randomly assign each image to 2 members of a panel of physicians who were trained in the World Health Organization CXR reading process. Each reader was masked to the case ID number, which contained a site identifier, and to the other reader’s interpretation. Reviewers were also prevented from interpreting CXRs from their own site [5]. The eCRF allowed reviewers to zoom, rotate, or resize the CXR image. The system identified discordant results and assigned these to a panel of adjudicators for final review. Digital chest auscultation sound files were transferred on a regular basis to the DCC from each site which participated in the digital auscultation substudy. Sound files were interpreted by a trained panel in a similar process to that used in the interpretation of CXRs. Maintaining these activities within the EDC system enabled real-time monitoring of progress of and results from these assessments throughout the study period.

### Training

Standardization of key PERCH clinical assessments and specimen collection procedures was achieved through on-site staff training, described in detail elsewhere [4].

DCC data managers demonstrated the PERCH EDC in webcasts and online training videos. A training version of the PERCH EDC system was provided for staff to practice entering mock data. New users were required to pass a brief quiz before receiving access to the live data system. During site visits and monthly Data Management Working Group calls, the Core team and DCC highlighted the available data management tools in the EDC system and reinforced the standardized interpretation and collection of data fields.

### Study Website

The secure study website contained current versions of printable CRFs, guidelines for completing each CRF, an EDC user’s guide, and documentation of form and system updates. SOPs were developed and made available on the study website for all study procedures and other processes that required specialized training. The study website also served as the portal to the EDC; this encouraged data entry staff to routinely view key study materials and communications from the Core team or DCC.

### Data Cleaning

Data cleaning occurred in 4 stages: (1) during data entry in response to detailed EDC system logic and range checks; (2) by site supervisor monitoring of EDC-automated reports of missing forms and fields; (3) by preprogrammed cross-form or complex checks performed bimonthly by the DCC; and (4) by inconsistencies identified by statistical analysts for data used in analyses.

The EDC checks and DCC query reports were supplemented by local data monitoring efforts to ensure staff members were adhering to study procedures. At all sites, lead investigators routinely reviewed DCC reports that tracked key data. At select sites, lead investigators downloaded data directly from the EDC system to perform internal data checks.

At the study start, the Core team and DCC staff reviewed all CRFs to identify potential errors that may occur both within and across forms. Priority variables required for primary and supporting analyses were identified through the development of detailed analysis plans. Data checks were prioritized based on the potential impact on primary study objectives. Operational data (eg, time each specimen arrived at the laboratory) or those anticipated to have limited impact on analyses were evaluated but excluded from query reports. Analyses were performed throughout the study to ensure availability, analyzability, and quality of all key data.

Sites were prompted to correct identified errors directly in the EDC system within 2 weeks of reporting. The EDC’s built-in audit history allowed study investigators to monitor frequency or rationale for data changes and the user who made the change.

### Study Progress and Performance Monitoring

**Figure 1.**
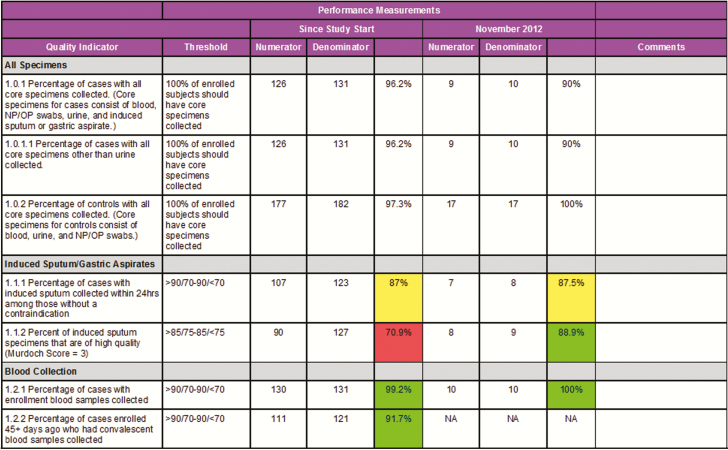
Excerpt from monthly quality indicator report. Green, meets expectations; yellow, requires investigation; red, immediate intervention required. Color-coding applied where defined performance thresholds varied from 100%. Abbreviations: NP/OP, nasopharyngeal/oropharyngeal.

The Core team, site PIs, and DCC staff defined detailed quality metrics to monitor study progress and to rapidly identify emerging trends in quality performance. These 50 unique quality criteria were used to summarize progress in study accrual, collection and handling of specimens, completion of follow-up assessments, and timely entry of CRFs into the EDC (Figure 1 and Supplementary Table 1). Performance measurements focused on the most critical indicators of internal study validity and often reflected multiple study procedures such that if the summary metric met performance criteria, the steps (eg, specimen handling and processing) required to achieve that metric must also have met expectations. The Core team reviewed monthly reports with site staff that were color-coded by performance (green = meets expectations; yellow = requires investigation; red = immediate action required) to troubleshoot logistical challenges or address need for retraining. Quality indicator reports were also shared across sites during operational and working group calls, which was effective in motivating sites to maintain or improve study quality.

Additional study progress reports evaluated trends in enrollment and availability of clinical and laboratory results by site and month of enrollment. The PERCH laboratory director [D. M.] verified reports of all individual and summary PCR and other key laboratory results. The Core team used operational reports to monitor progress in CXR and digital auscultation review processes and quality of the CXR images and sound files, and to evaluate standardization throughout the study period.

All reports were programmed in SAS and posted on the study website. Listings and other operational reports were automatically updated daily. Quality indicator reports and study progress reports were prepared monthly or as needed by the Core team.

### Standardization Across Analyses

To promote consistency in the use and interpretation of data across analyses, the Core team and DCC created analytic datasets containing key variables, including derived and recoded variables defined in analysis plans (eg, HIV infection and exposure status was derived from parental report of child’s status and maternal status, the child’s age, and test results). An EDC screen was designed to capture each derived variable, including name, definition, and programming code, and to automatically record any changes to the definitions or programming of these variables. More than 100 derived variables were required for the primary or supporting analyses. PERCH standard variable definitions were documented in data dictionaries and annotated CRFs that were shared across analysis teams.

## EVALUATION OF THE DATA MANAGEMENT SYSTEM, QUALITY, AND COMPLETENESS

Among the approximately 9000 cases and controls, analyzable laboratory results were available for 99% of blood cultures, nasopharyngeal/oropharyngeal PCR, and nasopharyngeal culture and 96% of whole blood PCR specimens (Table 1). Data were available for >94% cases and controls for each of the key demographic, clinical, vaccination, environmental, and risk factor variables (Table 2). Among all subjects, <0.001% of all required data points were missing and unexplained or out of expected range. More than 85% of participants had key laboratory tests performed and results available for analysis at the time of last subject enrollment. Of 4232 cases enrolled, only 11 (0.3%) were excluded from the primary etiology analysis because of missing or nonanalyzable data.

**Table 1.** Data Quality Summary: Percentage of Pneumonia Etiology Research for Child Health (PERCH) Cases and Controls With Specimens Collected and Test Results Available

Specimen and Results	Cases (n = 4232)		Controls (n = 5325)	
Blood culture specimens collected	4179	(98.7)	NA	NA
Culture results available—end of enrollment^a^	4161	(99.6)	NA	NA
Culture results available—final analysis^a^	4176	(99.9)	NA	NA
Whole blood specimens collected	4159	(98.3)	5145	(96.6)
PCR results available—end of enrollment^a^	3624	(87.1)	4562	(88.7)
PCR results available—final analysis^a^	3995	(96.1)	4987	(96.9)
NP/OP VTM specimens collected	4212	(99.5)	5311	(99.7)
PCR results available—end of enrollment^a^	3592	(85.3)	4502	(84.8)
PCR results available—final analysis^a^	4139	(98.3)	5199	(97.9)
NP STGG specimens collected	4175	(98.7)	5267	(98.9)
Culture results available—end of enrollment^a^	4160	(99.6)	5250	(99.7)
Culture results available—final analysis^a^	4172	(99.9)	5266	(100)

Data are presented as No. (%).

Abbreviations: NA, not applicable; NP, nasopharyngeal; OP, oropharyngeal; PCR, polymerase chain reaction; STGG, skim milk-tryptone-glucose-glycerin; VTM, viral transport medium.

^a^Percentages among those with specimens collected.

**Table 2.** Data Quality Summary, With Number and Percentage of Cases and Controls Missing Key Demographic and Risk Factor Fields

	Cases (n = 4232)		Controls (n = 5325)	
Characteristic	Data Field Missing	Data Field Unknown	Data Field Missing	Data Field Unknown
Demographic and clinical characteristics				
Age	0 (0.0)	0 (0.0)	0 (0.0)	0 (0.0)
Sex	0 (0.0)	0 (0.0)	0 (0.0)	0 (0.0)
Low birth weight or premature	16 (0.4)	20 (0.5)	14 (0.3)	13 (0.2)
Weight for age	0 (0.0)	13 (0.3)	6 (0.1)	18 (0.3)
HIV (South Africa and Zambia only)	0 (0.0)	3 (0.2)^a^	0 (0.0)	10 (0.6)^a^
Vaccination data				
Hib vaccine	2 (0.05)	165 (3.9)	5 (0.09)	142 (2.7)
PCV vaccine^b^	1 (0.03)	115 (4.0)	5 (0.2)	117 (3.7)
DTP vaccine	2 (0.05)	165 (3.9)	5 (0.09)	142 (2.7)
Environment and sanitation				
Crowding	27 (0.6)	13 (0.3)	16 (0.3)	1 (0.02)
Cooking fuel	21 (0.5)	3 (0.07)	17 (0.3)	4 (0.08)
Main source of drinking water	14 (0.3)	7 (0.2)	14 (0.3)	2 (0.04)
Toilet type	15 (0.4)	3 (0.07)	14 (0.3)	2 (0.04)
Breastfeeding				
Any breastfeeding	16 (0.4)	10 (0.2)	14 (0.3)	5 (0.1)
Duration of breastfeeding^c^	0 (0.0)	14 (0.4)	0 (0.0)	10 (0.2)
Household information				
Mother’s educational level	13 (0.3)	46 (1.1)	15 (0.3)	43 (0.8)

Data are presented as No. (%). “Data field missing” indicates a missing value; “Data field unknown” indicates a report of “Unknown” for a data point (ie, data available but not analyzable).

Abbreviations: DTP, diphtheria-tetanus-pertussis vaccine; Hib, *Haemophilus influenzae* type b; HIV, human immunodeficiency virus; PCV, pneumococcal conjugate vaccine.

^a^Composite variable of HIV status unknown because HIV serology or virological testing was not performed on these children.

^b^Restricted to sites using PCV during study: Kenya, The Gambia, Mali, and South Africa.

^c^Among children with breastfeeding.

In a survey of their experience with the PERCH EDC, the data leaders at the sites valued the design’s emphasis on data quality and reporting. Several commented that monthly quality indicator reports were especially important for monitoring trends in progress and potential need for additional staffing, training, or operational support. Importantly, sites’ success in meeting quality targets gave assurance of the validity of study data and value of standardization and training in complex procedures. Presenting interim results to the sites throughout enrollment helped to maintain motivation and engagement of staff in the study. One site investigator identified a need for comparable strategies and coordination support to be applied in clinical research programs in developing countries in an ongoing way.

## DISCUSSION

Successful conduct of the PERCH study demanded rigorous, collaborative approaches to data management and reporting. While this sort of oversight is typical for clinical trials, it is more unusual for observational studies, such as case-control studies, which do not have the same real-time data monitoring requirements as evaluations of interventions. The rigor with which PERCH was conducted assured a level of confidence in the data that is needed when misclassification and missing results can obscure or limit exploration of complex relationships between factors.

One measure of PERCH’s success is the completeness of analyzable data available for primary analyses, despite the volume of data (>20 CRFs) and complexity of study procedures. Among the approximately 9000 cases and controls, analyzable laboratory results were available from almost every child for almost every specimen.

A key advantage to PERCH data management was the capacity to maintain an internet-based data entry system at each site. This enabled site staff, the Core team at JHSPH, and the DCC staff to access study data in real time, to monitor data quality and study progress, and to respond quickly to address issues. Real-time access to laboratory results also informed selection of specimens for additional testing, and decisions on use of novel laboratory tests in the remainder of the study. Built-in data checks, query tools, and site-specific logic at the point of data entry prevented critical errors, simplified data entry, and significantly reduced data cleaning efforts that would have distracted staff away from other crucial study duties. This internet-based approach, while prioritizing data quality, was challenged by the relative complexity of initial site staff training and development of the system, and the need to establish reliable power supply and internet access at certain sites where these were not in place at the time PERCH began. Automated upgrades to internet browsers required the DCC to continuously test and adapt the PERCH data system to stay current. Slow local internet speeds during peak periods of activity limited the ability of several sites to rapidly resolve data queries at times. However, staff from the sites, DCC, and the Core team judged these limitations and challenges acceptable in light of the accessibility and flexibility of the online system.

An alternative, offline data system may have simplified study start-up, but risked shifting burden to the logistically challenging management of large volumes of paper forms. Previous large-scale international studies that used offline systems had to fax, ship or upload paper CRFs for data entry by off-site personnel less familiar with the data or for automated conversion into a central database. Several of these systems required costly and cumbersome printing and shipping of paper forms. Offline approaches also often resulted in delays of weeks or months in resolving missing data or errors, a timeline that would have exceeded the limited observation period for cases and controls in PERCH.

At all stages, data management effort was balanced among staff from sites, DCC, and Core team to prioritize accuracy and efficiency. Choices were made between where complexity was introduced, either at EDC system design, data entry, cleaning, coding, or analysis stages, favoring the approach that would result in the most reliable results overall. For example, sites opted to transfer PCR results files to the Core team and DCC for validation and conversion to analysis datasets outside of EDC. This approach required complex programming by the DCC to integrate PCR results with EDC data but allowed sites and laboratory leads to easily share and review results files and avoided transcription errors.

Site quality indicator reports and daily-updated clinical and laboratory reports were tools that successfully and quickly identified data errors or protocol deviations for resolution. To avoid overwhelming the sites and to maintain focus on study objectives, the burden of monitoring and resolving data queries was restricted to key clinical and laboratory data. Site staffing capacity was regularly assessed to maximize data quality within feasible limits.

The various PERCH approaches to data management depended on shared responsibility and successful collaboration among site PIs, site staff, study leaders, Core team coordinators, and the DCC staff. Balance was achieved between prioritizing study standardization and adapting data collection to accommodate diverse research environments and site settings. Tailored approaches to data checking and reporting emphasized the quality and completeness of critical study outcomes. The early, detailed planning and piloting of primary and supporting analyses guided prioritization of data management effort. The PERCH approaches may inform data management strategies of large-scale, multicountry clinical research observational studies of similar scope and complexity.

## Supplementary Data

Supplementary materials are available at *Clinical Infectious Diseases* online. Consisting of data provided by the author to benefit the reader, the posted materials are not copyedited and are the sole responsibility of the author, so questions or comments should be addressed to the author.

## Supplementary Material

Supplemental_Materials

## Notes

***Author contributions.*** N. L. W. and C. P. drafted the initial manuscript. M. D. K., A. J. D., M. M. H., D. E. P., D. R. M., and K. L. O. provided significant contributions to the manuscript. N. L. W., M. W., and M. S. led the data management team at the Data Coordinating Center with support from C. J. and O. N. K. L. O., O. S. L., D. R. M., and M. D. K. were involved in study design and supervised study conduct. A. N. D., J. O. A., D. G., E. H., L. H., A. K., L. K., D. P. M., U. O., D. P., P. S., P. S., A. S., K. S., P. S., and M. S. were involved in data collection and study conduction. All authors reviewed and approved the manuscript.

***Acknowledgments.*** We offer sincere thanks to the patients and families who participated in this study. We acknowledge the significant contributions of the PERCH Study Group and all PERCH investigators. We offer our gratitude to the members of the Pneumonia Methods Working Group and PERCH Expert Group for their time and lending expertise to assist the PERCH Study Group. See Supplementary Materials for a full list of names.

***PERCH Study Data Teams.*** Seydou Sissoko, Oualy Diawara, Moussa Fane, Dr Arif Uddin Shikder, Dr Sharifa Yeasmin, Dr Abu Sadat Md. Sayeem, Dr Farhana Parvin Flora, Dr Yasmin Jahan, Dr Mustafizur Rahman, Md. Sharhi BinElahi, Azwifarwi Mudau, Given Malete, Azwifarwi Mudau, Charné Combrinck, Lourens Combrinck, Grizelda Liebenberg, Lerato Qoza, Zunaid Kraft, Hendrick van Jaarsveld, Lerato Mapetla, Lisa-Marie Mollentze, Tsholofelo Mosome, Pattraporn Klanjatturat, Kazungu Siazeele, Magdalene Mwale, including many others who provided data entry and data management support at the sites throughout the study.

***Financial support.*** This work was supported by the Bill & Melinda Gates Foundation (grant number 48968 to the International Vaccine Access Center, Department of International Health, Johns Hopkins Bloomberg School of Public Health).

***Supplement sponsorship.*** This article appears as part of the supplement “Pneumonia Etiology Research for Child Health (PERCH): Foundational Basis for the Primary Etiology Results,” sponsored by a grant from the Bill & Melinda Gates Foundation to the PERCH study of Johns Hopkins Bloomberg School of Public Health, Baltimore, Maryland.

***Potential conflicts of interest.*** M. D. K. has received funding for consulting from Merck, Pfizer, and Novartis, and grant funding from Merck. All other authors report no potential conflicts. K. L. O. has received grant funding from GSK and Pfizer and participates on technical advisory boards for Merck, Sanofi Pasteur, PATH, Affinivax, and ClearPath. All authors have submitted the ICMJE Form for Disclosure of Potential Conflicts of Interest. Conflicts that the editors consider relevant to the content of the manuscript have been disclosed.
