# Supplementary material for: Data Management and Data Quality in PERCH, a Large International Case-Control Study of Severe Childhood Pneumonia
Source: Clin Infect Dis. 2017 May 27;64(Suppl 3):S238–44. doi: 10.1093/cid/cix080 (PMC5447839; doi:10.1093/cid/cix080)
Supplement: Supplemental_Materials [file cix080_suppl_Supplemental_Materials.pdf]

**Appendix Table 1. PERCH Quality Indicators that were evaluated monthly for each site**

| |
|---|
| **All Specimens** |
| 1.0.1 Percentage of cases with all core specimens collected. (Core specimens for cases consist of blood, NP/OP swabs, urine, and induced sputum or gastric aspirate.) |
| 1.0.1.1 Percentage of cases with all core specimens other than urine collected. |
| 1.0.2 Percentage of controls with all core specimens collected. (Core specimens for controls consist of blood, urine, and NP/OP swabs.) |
| **Induced Sputum/Gastric Aspirates** |
| 1.1.1 Percentage of cases with induced sputum collected within 24hrs among those without a contraindication. |
| 1.1.2 Percent of induced sputum specimens that are of high quality. |
| **Blood Collection** |
| 1.2.1 Percentage of cases with enrollment blood samples collected. |
| 1.2.2 Percentage of cases enrolled 45+ days ago who had convalescent blood samples collected. |
| 1.2.3.1 Percentage of cases with 30-day follow-up completed who had convalescent blood samples collected. |
| 1.2.4 Percentage of controls with enrollment blood sample collected. |
| 1.2.5 Percentage of blood cultures that were contaminated. |
| 1.2.6 Percentage of blood cultures that were collected before antibiotics were administered at study hospital. |
| **NP/OP Swabs** |
| 1.3.1 Percentage of cases with NP flocked, NP rayon, and oral swabs collected. |
| 1.3.2 Percentage of controls with NP flocked, NP rayon and oral swabs collected. |
| **Lung Aspirate** |
| 1.4.1 Percentage of cases whose CXR indicates eligible for lung aspirate, who have had lung aspirate collected (for sites collecting lung aspirate specimens). |
| 1.4.2 Percentage of lung aspirates that yielded a bacterial pathogen by culture. |
| **Post Mortem Specimen** |
| 1.5.1 Percentage of fatal cases that had a post mortem specimen collected <= 4hrs after death (for sites collecting post mortem specimens). |
| **Urine** |
| 1.6.1 Percentage of cases that had urine specimens collected. |
| 1.6.2 Percentage of controls that had urine specimens collected. |
| **CXR Quality Indicators** |
| 1.7.1 Percentage of cases with chest x-ray done. |
| 1.7.2 Percentage of films that were sufficient quality for a radiologist reading. |

| |
|---|
| **Follow Up Assessments** |
| 1.8.1 Percentage of cases discharged 24+ hrs after enrollment who have had 24hr limited clinical assessment completed. |
| 1.8.2 Percentage of cases discharged 48+ hrs after enrollment who have had 48hr limited clinical assessment completed. |
| 1.8.3 Percentage of cases admitted >30 days ago whose Case Discharge form indicates discharge alive, who have had 30 day vital status follow up form submitted. |
| 1.8.4 Percentage of cases admitted >30 days ago whose Case Discharge form indicates discharge alive, who have had 30 day follow up interview conducted. |
| **Case logs and enrollment yield** |
| 2.0.1 Percentage of patients meeting pre-screening criteria who are screened for PERCH. |
| 2.0.2 Percentage of eligible severe pneumonia patients enrolled. |
| 2.0.3 Percentage of eligible very severe pneumonia patients enrolled. |
| **Control logs and enrollment yield** |
| 3.0.1 Percentage of minimum controls enrolled in the last 30 days. |
| **Specimen transport and handling** |
| 4.0.1 Percent of Blood culture bottles inoculated and put into incubator within 24 h. |
| 4.0.2 Percent of NP flocked + NP rayon + OP swabs collected and transported to laboratory within 8 h: Cases. |
| 4.0.3.1 Percent of NP flocked + NP rayon + OP swabs collected and transported to laboratory within 8 h: Controls. |
| 4.0.4 Percent of IS specimens collected and transported to laboratory within 24 h. |
| 4.0.5 Percent of gastric aspirate specimens collected and transported to laboratory within 96 h. |
| 4.0.6 Percent of lung aspirate specimens collected and transported to laboratory within 24 h. |
| 4.0.7 Percent of pleural fluid specimens collected and transported to laboratory within 24 h. |
| 4.0.8 Percent of NA extractions completed within 3 days of EDTA collection. |
| **Laboratory QA/QC** |
| 5.0.1 Percent of clinical specimens rejected upon receipt at the laboratory (for reasons such as mislabeling, leakage, inappropriate transport etc.). |
| 5.0.2 Pneumococci isolated from blood cultures. |
| 5.0.3 Average volume of induced sputum specimen. |
| **Blood volumes** |
| 5.1.1 Percent of blood culture bottles for which adequate volume for weight is obtained (1 mL if child's weight <= 1 kg; 2 mL if child's weight > 1 kg). |
| 5.1.2 Percent of cases with adequate volume of blood collected in EDTA-plasma tube #1 (0.5 mL expected). |
| 5.1.2.1 Percent of cases with adequate volume of blood collected in EDTA-plasma tube #2 (1 mL if child's weight < 3 kg; 1.5 mL if child's weight >= 3 kg). |
| 5.1.3 Percent of controls with adequate volume of blood collected in EDTA-plasma tube #1 (2 mL expected). |

| |
|---|
| 5.1.4 Percent of cases with adequate volume of blood collected in plain/red-top tube (0.5 mL if child's weight <= 1 kg; 1 mL if child's weight > 1 kg). |
| 5.1.5 Percent of controls with adequate volume of blood collected in plain/red-top tube (2 mL expected). |
| 5.1.6 Percent of cases for which adequate convalescent (follow-up) serum volume is obtained (2 mL expected). |
| 5.2.1 Percent of cases with a Pneumococcal positive blood culture who have serotype data available. |
| **Reporting of data and feedback plans** |
| 6.0.1 Number of CRF 01s entered into EDC within one week of subject enrollment. |
| 6.0.2 Percent of key clinical and lab CRFs entered into EDC within 2 weeks of subject enrollment. |

**Acknowledgments:**

***PERCH Study Group***. **Johns Hopkins Bloomberg School of Public Health, Baltimore, Maryland**: Katherine L. O'Brien (PI), Orin S. Levine (Former PI, current affiliation Bill & Melinda Gates Foundation, Seattle, Washington**),** Maria Deloria Knoll (co-PI), Daniel R. Feikin (joint affiliation with Centers for Disease Control and Prevention, Atlanta, Georgia), Andrea N. DeLuca, Amanda J. Driscoll, Nicholas Fancourt, Wei Fu, Laura L. Hammitt, Melissa M. Higdon, E. Wangeci Kagucia, Ruth A. Karron, Mengying Li, Daniel E. Park, Christine Prosperi, Zhenke Wu, Scott L. Zeger; **The Emmes Corporation, Rockville, Maryland:** Nora L. Watson, **Nuffield Department of Clinical Medicine, University of Oxford, United Kingdom**: Jane Crawley; **University of Otago, Christchurch, New Zealand**: David R. Murdoch; **ICDDR, b, Dhaka and Matlab, Bangladesh**: W. Abdullah Brooks (site PI), Hubert P. Endtz, Khalequ Zaman, Doli Goswami, Lokman Hossain, Yasmin Jahan, Hasan Ashraf; **Medical Research Council, Basse, The Gambia**: Stephen R. C. Howie (site PI), Bernard E. Ebruke, Martin Antonio, Jessica McLellan, Eunice Machuka, Arifin Shamsul, Syed M.A. Zaman, Grant Mackenzie; **KEMRI-Wellcome Trust Research Programme, Kilifi, Kenya**: J. Anthony G. Scott (site PI and PERCH co-PI), Juliet O. Awori, Susan C. Morpeth, Alice Kamau, Sidi Kazungu; **Division of Infectious Disease and Tropical Pediatrics, Department of Pediatrics, Center for Vaccine Development, Institute of Global Health, University of Maryland School of Medicine, Baltimore, Maryland and Centre pour le Développement des Vaccins (CVD-Mali), Bamako, Mali**: Karen L. Kotloff (site PI), Milagritos D. Tapia, Samba O. Sow, Mamadou Sylla, Boubou Tamboura, Uma Onwuchekwa, Nana Kourouma, Aliou Toure; **Respiratory and Meningeal Pathogens Research Unit, University of the Witwatersrand , Johannesburg, South Africa**: Shabir A. Madhi (site PI), David P. Moore, Peter V. Adrian, Vicky L. Baillie, Locadiah Kuwanda, Azwifarwi Mudau, Michelle J. Groome, **Thailand Ministry of Public Health – U.S. CDC Collaboration, Nonthaburi, Thailand**: Henry C. Baggett (site PI), Somsak Thamthitiwat, Susan A. Maloney (former site PI), Charatdao Bunthi, Julia Rhodes, Pongpun Sawatwong, Pasakorn Akarasewi (site co-PI, Ministry of Public Health); **Boston University School of Public Health, Boston, Massachusetts and University Teaching Hospital, Lusaka, Zambia**: Donald M. Thea (site PI), Lawrence Mwananyanda, James Chipeta, Phil Seidenberg, James Mwansa, Somwe wa Somwe, Geoffrey Kwenda. **Canterbury Health Laboratory, Christchurch, New Zealand:** Trevor P. Anderson, Joanne Mitchell.

***PERCH Expert Group.*** William C. Blackwelder, Harry Campbell, John A. Crump, Adegoke Falade, Menno D de Jong, Claudio Lanata, Kim Mulholland, Shamim Qazi, Cynthia G. Whitney.

***Pneumonia Methods Working Group.*** Robert E Black, Zulfiqar A Bhutta, Harry Campbell, Thomas Cherian, Derrick W Crook, Menno D de Jong, Scott F Dowell, Stephen M Graham, Keith P Klugman, Claudio F Lanata, Shabir A Madhi, Paul Martin, James P Nataro, Franco M Piazza, Shamim A Qazi, and Heather J Zar.

***PERCH Contributors:***

**Bangladesh:** Kamrun Nahar, Arif Uddin Sikdir, Sharifa Yeasmin, Dilruba Ahmed, Muhammad Ziaur Rahman, Muhammad Yunus, Muhammad Al Fazl Khan, Muhammad Jubayer Chisti, Abu Sadat Muhammad Sayeem, Shahriar Bin Elahi, Mustafizur Rahman; **The Gambia:** Michel Dione, Emmanuel Olutunde, Peter Githua, Ogochukwu Ofordile, Rasheed Salaudeen, David Parker; **Kenya:** Shebe Mohamed, Siti Ndaa, Micah Silaba, Neema Muturi, Angela Karani, Sammy Nyongesa, Anne Bett, Daisy Mugo, Salim Mwarumba, Robert Musyimi, Andrew Brent, James Nokes, David Mulewa, Joyce Sande, John Odhiambo, Joshua Wambua, Nuru Kibirige, Caroline Mulunda, Hellen Mjalla, Norbert Katira, Karen Dama, Loice Masha, Christine Mutunga, Mwanajuma Ngama, Stephen Mangi, Riziki Anthony, Mwarua Yubu, Elijah Wakili, Benson Katana, Shoboi Mgunya, Emmanuel Mumba, Benedict Mver, George Kuria, Felix Githinji, Norbert Kihuha, Boniface Jibendi, Tahreni Bwanaali, Agustus Kea; **Mali:** Nana Kourouma, Aliou Toure, Mahamadou Diallo, Breana Barger-Kamate, Mariam Samake, Seydou Sissoko, Abdoul Aziz Maiga, Mariam Samake, Toumani Sidibe, Mariam Sylla, Aziz Diakite, Bassirou Diarra; **South Africa:** Azwidihwi Takalani, Andrea Hugo, Susan Nzenze, Ndulela Titi, Mmabatho Selela, Malebo Motiane, Minah Nkuna, Nonhlanhla Tsholetsane, Sibonsile Moya, Debra Katisi, Tondani Netshishivhe, Lerato Mapetla, Gudani Singo, Simphiwe Gasa, Cece Mgenge, Nozipho Mthunzi, Nombulelo Monedi, Tanja Adams, Shafeeka Mangera, Jeannette Wadula, Peter Tsaagane, Jenifer L. Vaughan, Sakina Loonat, Martin Hale, Sugeshnee Pather, Mariëtte Middel, Siobhan Trenor, Palesa Morailane, Ntombi Maya, Rene Sterley, Charné Combrinck, Given Malete, Lerato Qoza, Grizelda Liebenberg, Hendrik van Jaarsveld, Zunaid Kraft, Lisa-Marie Mollentze, Lourens Combrinck, Tsholofelo Mosome; **Thailand:** Sununta Henchaichon, Dr. Tussanee Amornintapichet, Dr. Somchai Chuananont, Toni Whistler, Juraiporn Ratanodom, Patranuch Sapchookul, Ornuma Sangwichian, Sirirat Makprasert, Manoon Hirunsalee, Possawat Jorakate, Anek Kaewpan, Duangkamol Siludjai, Apiwat Lapamnouysup, Dr. Wantana Paveenkittiporn, Waraporn Ubonphen, Dr. Peera Areerat, Ms.Yupapan Wannachaiwong, Ms Tewa Faipet, Ms Punnat Natnarakorn, Ms Ahchanan Sacharone, Mr.Winai Makmool, Ms. Kanlaya Sornwong, Ms. Promporn Sansuriwong, Ms. Ratchanida Potiya, Ms. Wasana Hongsawong, Ms.Wipa Matchaikhen, Ms. Thatsanawan Chaiyabil, Ms.Piyapai Wannarach, Ms Chamaiporn Wadeesirisak, Mr. Yuttapong Norapet, Mattana Bangkung, Mr. Barameht Piralam, Sathapana Naorat, Anchalee Jatapai, Prasong Srisaengchai, Dr. Leonard Peruski, Ms.Dawan Phaensoongnoen, Ms.Tussaaorn Klangprapan,

Ms.Narawadee Dumrongdee, Ms.Atchara Srithongkham, Mr. Piyawut Noinont, Ms. Pornthip Kamlee, Ms.Siyapa  Mongkornsuk; **Zambia:** Justin Mulindwa, Musaku Mwenechanya, John Mwaba, Magdalene Mwale, Julie Duncan, Kazungu Siazele, Muntanga Mapeni, Emily Hammond; **Canterbury Health Laboratory, Christchurch, New Zealand:** Rose Watt, Shalika Jayawardena.
